# Landscape Diversity Related to Buruli Ulcer Disease in Côte d'Ivoire

**DOI:** 10.1371/journal.pntd.0000271

**Published:** 2008-07-30

**Authors:** Télesphore Brou, Hélène Broutin, Eric Elguero, Henri Asse, Jean-François Guegan

**Affiliations:** 1 Université d'Artois, Faculté d'Histoire-Géographie, Arras, France; 2 Génétique et Evolution des Maladies Infectieuses, GEMI-UMR 2724 IRD-CNRS, Equipe “Dynamique des Systèmes et Maladies Infectieuses”, Montpellier, France; 3 Institut Raoul Follereau d'Adopzé, Université de Cocody, UFR des Sciences Médicales, Abidjan, Côte d'Ivoire; University of Tennessee, United States of America

## Abstract

**Background:**

Buruli ulcer disease (BU), due to the bacteria *Mycobacterium ulcerans*, represents an important and emerging public health problem, especially in many African countries. Few elements are known nowadays about the routes of transmission of this environmental bacterium to the human population.

**Methodology/Principal Findings:**

In this study, we have investigated the relationships between the incidence of BU in Côte d'Ivoire, western Africa, and a group of environmental variables. These environmental variables concern vegetation, crops (rice and banana), dams, and lakes. Using a geographical information system and multivariate analyses, we show a link between cases of BU and different environmental factors for the first time on a country-wide scale. As a result, irrigated rice field cultures areas, and, to a lesser extent, banana fields as well as areas in the vicinity of dams used for irrigation and aquaculture purposes, represent high-risk zones for the human population to contract BU in Côte d'Ivoire. This is much more relevant in the central part of the country.

**Conclusions/Significance:**

As already suspected by several case-control studies in different African countries, we strengthen in this work the identification of high-risk areas of BU on a national spatial scale. This first study should now be followed by many others in other countries and at a multi-year temporal scale. This goal implies a strong improvement in data collection and sharing in order to achieve to a global picture of the environmental conditions that drive BU emergence and persistence in human populations.

## Author Summary

Buruli ulcer (BU) is one of the most neglected but treatable tropical diseases. The causative organism, *Mycobacterium ulcerans*, is from the family of bacteria that causes tuberculosis and leprosy. This severe skin disease leads to long-term functional disability if not treated. BU has been reported in over 30 countries mainly with tropical and subtropical climates, but Côte d'Ivoire is one of the most affected countries. *M. ulcerans* is an environmental bacterium and its mode of transmission to humans is still unclear, such that the disease is often referred to as the “mysterious disease” or the “new leprosy”. Here, we explored the relationship between environmental and socioeconomic factors and BU cases on a nationwide scale. We found that irrigated rice field cultures areas, and, to a lesser extent, banana fields as well as areas in the vicinity of dams used for irrigation and aquaculture purposes, represent high risk zones for the human population to contract BU in Côte d'Ivoire. This work identifies high-risk areas for BU in Côte d'Ivoire and deserves to be extended to different countries. We need now to obtain a global vision and understanding of the route of transmission of *M. ulcerans* to humans in order to better implement control strategies.

## Introduction

Buruli ulcer is a severe human skin disease caused by *Mycobacterium ulcerans*. It represents now the third mycobacterial infection in the world behind tuberculosis due to *M. tuberculosis* and leprosy, caused by *M. leprae*. The first clinical description of the disease agent was done in Australia in 1958 [1], even though disease cases have been recorded since the end of the XIX^th^ century in Uganda, in the Buruli area [2]. During the last decades, a dramatic extension of the spatial distribution of Buruli ulcer disease as well as increase of the number of infected people has been reported in many parts of the world. Highest incidences are now observed in Western Africa with 20,000, 6,000 and 4,000 cases observed in 2005 in Côte d'Ivoire, Ghana and Benin, respectively [1],[3],[4].

*Mycobacterium ulcerans* is an environmental bacterium and its mode of transmission to humans is still unclear, this is why the disease is often referred to as the “mysterious disease” or the “new leprosy”. Recent findings on the life cycle of the Buruli ulcer's agent have enhanced current evidence on several points. First, it has been shown that *M. ulcerans* can develop as biofilms on the surface of aquatic plants [5]. More specifically, some freshwater aquatic plants might be involved in the mycobacterium life-cycle as potential intermediate hosts or trophic chain concentrators [5]–[7]. Secondly, contrasted animal species were found infected by the bacterium in natural conditions, e.g. fishes, frogs [8]–[9] or koalas (see [10] for review). Recently, an impressive field study also generated additional environmental data regarding *M. ulcerans* in nature [11]. Third, aquatic insects are also suspected to act as vector and to transmit the disease by biting. It has been demonstrated that *M. ulcerans* is present in salivary glands of African water bugs of the family *Naucoridae*, and that infected water bugs could transfer the pathogen to mice [6],[12],[13]. Infected insects were also found in endemic areas. Finally, it is generally admitted by medical and scientific communities that specific environmental niches, which still need to be precisely defined, favour the occurrence of the disease [11], [14]–[18]. Based on several case-control studies performed in different African countries, freshwater ecosystems like rivers, man-made ponds and lakes, or marshy zones and irrigated perimeters represent risk factors to BU [19]–[21]. At nation-wide or regional scale, other studies also showed relations between BU infections and different environmental factors [22]–[24], as for instance landscape cover attributes [25] or arsenic in water [26] in Ghana. Despite these specific studies, Buruli ulcer is still a mysterious disease and all new findings will contribute to help national and international public health authorities to fight this highly deleterious pathogen and neglected disease. For this reason, all information on the relations between the environment and the disease occurrence are highly relevant for a better understanding of the disease as a whole.

Here we propose to perform a first nation-wide scale study in Côte d'Ivoire of the link between environmental but also socio-economic factors and Buruli ulcer cases, based on spatial mapping and multivariate statistical analyses. First detection of BU in Côte d'Ivoire occurred in 1981 but the number of cases clearly increased in 1987 and became then a national public health problem [20].

## Methods

### Epidemiological data

The Buruli ulcer notifications database was generated by the Institute Raoul Follereau in Côte d'Ivoire. All notified cases were diagnosed by medical doctors from the institution based mainly on clinical signs and sometimes confirmed also by tissue biopsy or ulcer swab examination. Data correspond to annual cases reports from primary healthcare centers and central hospitals distributed all over the national territory in 1997. The central hospitals serve towns and cities with more than 5,000 inhabitants. In Côte d'Ivoire, up to 54 percent of the human population lives in a town with a primary health care center or hospital; and 91 percent lives less than 15 km from such a town [27]. Permission to use these data for the present study has been granted by the National Program for Buruli ulcer Control from the Ministry of Health and Public Hygiene of Côte d'Ivoire (Pr H. Asse).

### Environmental and socio-economic data

**Figure 1:**
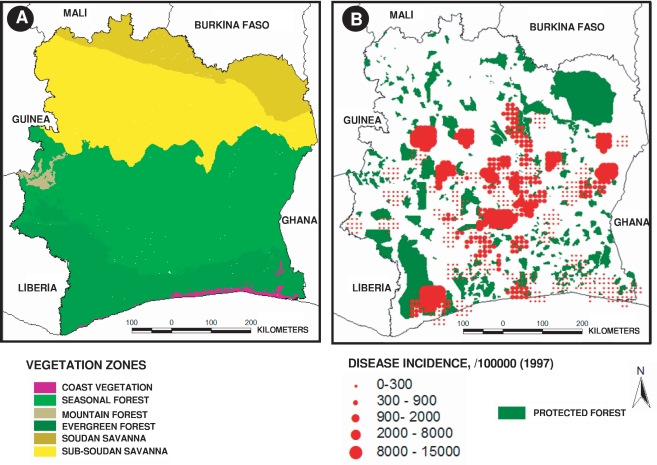
Map of (A) large ecosystem vegetation zones, and (B) primary forest patches on the geographical distribution of Buruli ulcer cases in Côte d'Ivoire in 1997.

Environmental data correspond to vegetation types and surfaces (forest or cultivated areas) and dams (location and superficies). Data concerning vegetation were extracted from two sources. To provide an overview of the global vegetation zones in the country, we first generated a map (Figure 1A) based on vegetation map by Guillaumet [28] which presents the principal zones of vegetation based on 1979 aerial photographs (50 centimeter resolution). Secondly, in order to obtain the most realistic information about forest patches corresponding to the BU reported period, we used the map of forest reservation in Côte d'Ivoire from the National Bureau of Technical and Development Studies [29] representing the recent state of the forest cover, based on 1993 and updated with 2000 SPOT images (20 meters resolution) to build a second map representing the current state of forest distribution in the country (Figure 1B). This recent map also enabled us to compute detailed geometrical characteristics (distances and areas) used in statistical analyses.

Côte d'Ivoire is characterized by some well-known contrasting ecosystems (Figure 1A): areas of wet dense forest and bush extending roughly South of the 8°N latitude line, and dry forest and Sudanian savanna ecosystems in the Northern region of the country. The origin of this division is primarily climatic, since the transition from wet dense forest to savanna-like ecosystems is associated with the change from four climatic equatorial seasons in the South to two tropical seasons in the North [28].

Data concerning dams were retrieved at the Côte d'Ivoire National Centre for mapping and remote-sensing, at the University of Abidjan-Cocody, by one of the authors (TB). They correspond to the dams' geographical position and total surface area for the year 1997. There are over 500 dams in Côte d'Ivoire and the majority of them were built between 1970 and 1990 in order to allow the cultivation of water-demanding crops, especially in the Central region and more specifically in the Northern region, where rainfall is not sufficient to sustain this kind of production. These water bodies have facilitated the establishment of irrigated rice cultures, and they are also a source of freshwater for cattle and fish farms.

Regarding socio-economic parameters, agricultural data were provided by the Côte d'Ivoire Ministry of Agriculture. They concerned *i*) the type of cultivated areas, and *ii*) the crop production (in tons) for the year 1997 for each of the 54 prefectures. For the present study, we only considered rice and banana fields, which require a constant high humidity and intensive water supply. Thus we did not consider crops which do not require irrigation, such as cassava ground nut and corn.

### Analysis methods

**Figure 2:**
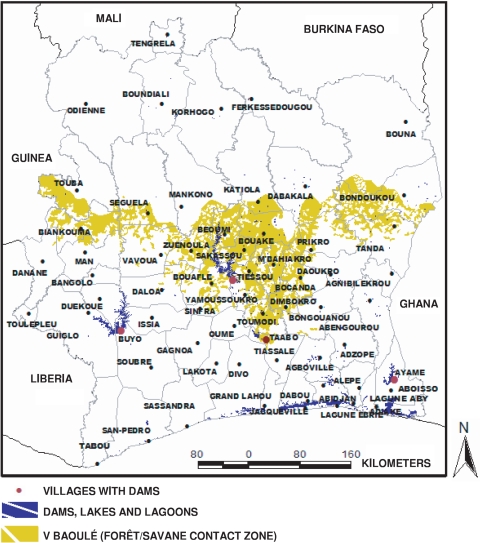
Préfectoral limits and main villages.

**Figure 3:**
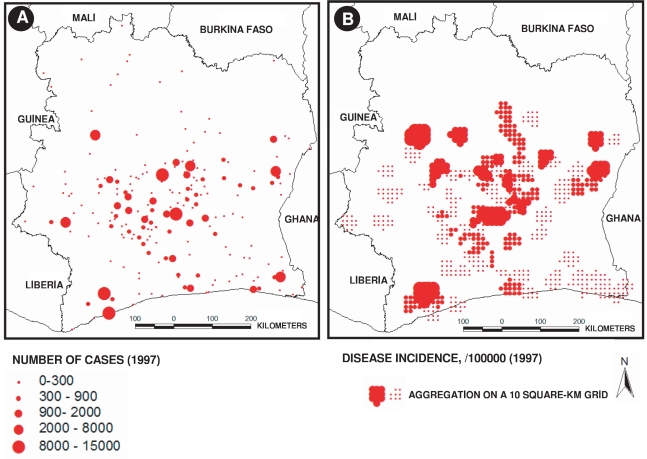
Geographical distribution of Buruli ulcer cases in Côte d'Ivoire in 1997. (A) raw incidence data (per 100,000 inhabitants), and (B) aggregated incidence on a 10 km grid-square.

**Figure 4:**
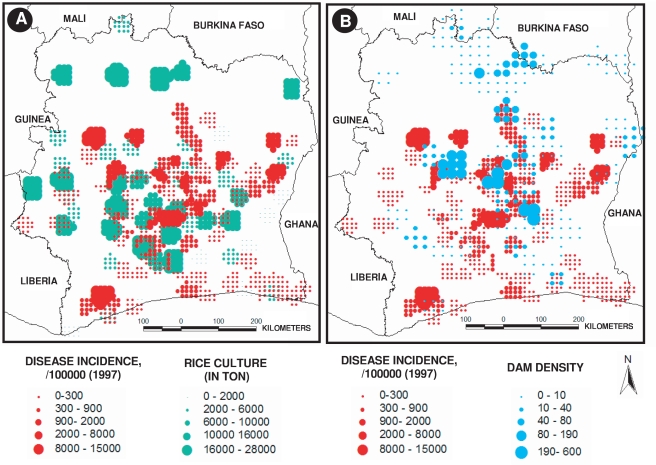
Map of (A) rainy and seasonal rice field cultures, and (B) dam density distribution and geographical distribution of Buruli ulcer cases in Côte d'Ivoire in 1997.

Epidemiological data correspond to cases detected at hospital or primary healthcare centers situated in each prefecture (Figure 2). Raw disease case data (Figure 3A) were transformed to numbers of cases per 100,000 inhabitants, considering the whole population of the prefecture of each 202 hospitals or health care centers. Moreover, it is known that each inhabitant lives in an average 10 km distance from a hospital in Cote d'Ivoire [27]. For this reason, we interpolated our epidemiological data on a regular 10 km×10 km grid-square by the inverse distance weighting method, using the Surfer software [30],[31] (Figure 3B). In order to illustrate environmental data, we generated maps representing both epidemiological and environment data by interpolating environment point data (i.e. dams and rice crops data) on the same regular grid (Figures 1 and 4). However, we did not use these interpolated data but the raw data for the following statistical analysis.

Surface data concerning forest area and vegetation zones were not transformed. The surface areas for the different types of ecosystems studied, e.g., evergreen forest patches and rice-field cultures were transformed according to the percentage of surface coverage on the total prefecture surface area size in km-square.

We used Geographical Information System (GIS) to explore the relationships between environmental and epidemiological data [32]. In this analysis, we used the original data instead of the interpolated data. We first arbitrarily defined 4 influence-by-distance classes (i.e., <5 km, 5–10 km, 10–15 km, 15–20 km) for Buruli ulcer disease occurrence, which corresponded to different distances between a given case and the nearest hydro-agricultural dam or the nearest forest patch respectively. Based on this distribution, we analysed the variation of the rate of disease incidence according to the different distances for the two environmental parameters under scrutiny, i.e., dams or forest patches. We thus considered here the location of dams and forest patches but not their surface. Concerning dams, we also made a regional study distinguishing three major hydrological regions: the Kossou region in the Centre of country, the Buyo region in the West, and the Southern region. Linear trends were tested by using simple linear model.

Finally, we used logistic regression [33] to analyse the statistical relationships between hydro-agricultural environmental conditions and incidence of Buruli ulcer disease by administrative division. The explicative variables considered were: forest surface area (in hectare), irrigated rice production (in thousands of tons), banana production (in thousands of tons), and dam surface area (in hectare). To make the analysis more complete, i.e., to better capture the multivariate dimension of the environmental conditions on disease emergence, we integrated the two-factors interaction terms.

## Results

### Ecosystem types and Buruli ulcer disease; mapping approach

Figure 3 illustrates Buruli ulcer cases and the aggregated distribution of incidences on a 10 km grid-square, in Côte d'Ivoire for the year 1997. Southern Côte d'Ivoire, rich in evergreen forest and forest/savanna-like landscapes (Figure 1A), had a very high Buruli ulcer incidence (more than 5,000 infected people for 100,000 inhabitants), whereas we observed a very low incidence in the North (less than 10 infected people for 100,000 inhabitants). In particular, the highest incidence (more than 10,000 cases for 100,000 inhabitants) was near the contact zone between forest and savanna, notably in proximity of the “V-Baoulé” region in the centre of Côte d'Ivoire. Figure 1B also illustrates the spatial relationship between Buruli ulcer cases and evergreen forest patches.

### Hydro-agricultural features and Buruli ulcer disease

**Figure 5:**
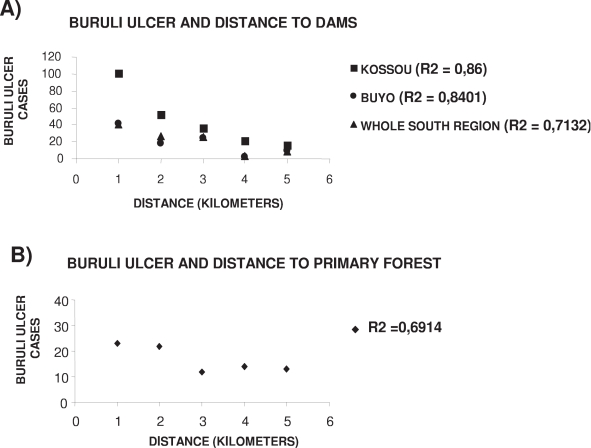
Buruli ulcer cases in relation with distance to (A) dams and (B) primary forest.

Figure 4A shows that the rice field areas exhibited the highest rate of disease notifications, notably near the large lakes in the Centre of the country, an area which provides nearly 40 per cent of the national rice production [34]. Together with the regions of high rice production, highest incidence spots of Buruli ulcer disease notification were mainly located in areas with a high density of dams (Figure 4B). It was particularly true for the area of the great lakes, in the Centre of the country, which hosts more than a half of the country's hydro-agricultural equipments, with an average reservoir surface area of 2 sq-km. When considering the geographical distance between Buruli ulcer disease cases and distance to dams or forest patches, we found significant negative relationships (Figure 5). In the Kossou region, located in the central part of Côte d'Ivoire, Buruli ulcer disease incidence was up to 100 notifications per 100,000 inhabitants within a 5 km-distance radius from the closest reservoir, but it dropped to 40 cases for a 5–10 km-distance, and to less than 20 cases for a 20 km-distance (r^2^ = 0.86, *P*<0.01; Figure 5A). Concerning the Buyo region in the West, and the whole Southern region, the relationship between Buruli ulcer incidence and distance to dams was also significant (r^2^ = 0.84, *P*<0.01; and r^2^ = 0.71, *P*<0.01, respectively; Figure 5). We observed the same negative relationship between disease incidence and increasing distance from a primary forest patch (r^2^ = 0.69, *P*<0.01; Figure 5B).

### Environmental risk factors for Buruli ulcer disease in Côte d'Ivoire

Table 1 illustrates the main results from the logistic regression analysis performed to explain Buruli ulcer disease incidence. This analysis showed that both the irrigated rice crops (*P*<0.001) and, to a lesser extent, the forest patch surface (*P* = 0.001) could increase the BU risk within communities by 77.5% and 3%, respectively. In contrast, the dam surface is clearly associated with a lower risk of BU infection (*OR* equals 0.74) even if we previously showed a significant positive correlation between BU and distance to dams (Figure 5). This apparent discrepancy can easily be explained by the different effects of the presence and the use by populations (contact with population) of dams, as discussed below.

**Table 1:** Values of *OR* (Odds Ratio) and *P* (probability) obtained from the logistic model to explain Buruli ulcer case notifications in Côte d'Ivoire, western Africa.

Variables	*OR*	Confidence Interval (95%)		*P*
		Lower value	Upper value	
Forest surface area (FSA), in hectare	1,0339	0,9958	1,0735	**0,000044**
Irrigated rice crops (IRP), in 1000 tons	1,7752	1,6748	1,8816	**<10^−6^**
Banana crops (BP), in 1000 tons	0,9773	0,8927	1,0699	0,595728
Dam surface area (DSA), in hectare	0,7478	0,6763	0,8269	**<10^−6^**
FSA×IRP interaction	0,999	0,9901	1,008	0,021864
FSA×BP interaction	1,0067	0,9992	1,0142	**0,000934**
DSA×BP interaction	1,0588	0,9914	1,1309	**0,000659**
IRP×BP interaction	0,8938	0,8398	0,9514	**<10^−6^**
IRP×DSA interaction	1,2507	1,0797	1,4489	**0,001217**
FSA×DSA interaction	1,0171	0,896	1,1547	0,793815

See both the Methods and Results sections for further details.

However, since interactions were included in the model, these results are to be interpreted with care. Main effects are to be interpreted as odds-ratios for one covariate when all other covariates are fixed at their base value. For instance, the value 0.75 for dam area means that surface of dams is associated with a reduced Buruli ulcer incidence, *only in places where there is no rice, no banana, etc*. When rice is present, the odds-ratio for an increase of one unit in dam area is equal to 0.75×1.25 = 0.94 (with 1.25 corresponding to the OR value for the interaction between both parameters), not significant. The odds-ratio for a 1,000 ton increase in rice production and an increase of dam area with respect to no-rice is 1.77×0.75×1.25 = 1.66.

In reality, the different habitats are superimposed in space (see Figures 1 and 4), an observation which was in accordance with the statistical results of the influence of interactions, and, thus, could not be considered as specific variables. Two-way interaction terms between the forest patch coverage-banana crops and the dam surface-banana crops variables also appeared marginally significant to explain the increase of BU in Côte d'Ivoire, with odds-ratio 1.0067 (*P*<0.001; near 0.6%) and 1.0588 (*P*<0.001; near an order of 6%), respectively. Finally, the irrigated rice crops versus the banana crops interaction term was a significant protective factor (*OR* = 0.8938, *P*<10^−6^) in the present study.

## Discussion

Infectious agents indirectly transmitted to or between humans because of human-modified environments account for many emerging and re-emerging non-zoonotic or zoonotic diseases today [35]. The list of emerging diseases associated with human behaviours and environmental perturbations is still increasing [36],[37]. Concerning Buruli ulcer disease, although humans are dead-end hosts for the causative agent, *Mycobacterium ulcerans*, it is well known that the risk of infection is greatly increased by marked exposure to aquatic environments [38]. Even though the disease agent life-cycle is poorly understood, there is a consensus within the public health and scientific community that human behavior, i.e. frequentation of freshwaters for professional or recreational activities, and environmental aquatic alterations are major disease risk factors [19]. However, an extensive quantitative analysis of disease risk is dramatically lacking for Buruli ulcer [39], and risk factor analyses, like the one described in this paper, in parallel to the understanding of the evolutionary properties of the pathogenic microorganism, are definitely necessary to circumvent and control the spread of the disease.

Based on a country-wide statistical investigation on Buruli ulcer cases in Côte d'Ivoire, we have observed the highest incidence of Buruli ulcer disease in the Centre of Côte d'Ivoire, and more particularly in the sector of “V Baoulé”, where there is also the highest concentration of hydro-agricultural dams (the majority of the villages are located less than 5 km from a dam), used for the development of agriculture, especially semi-intensive rice-field and banana production. Interestingly, high occurrence of Buruli ulcer disease was also notified in the South-Western region of the country, where there is a very low density of man-made dams, but where environmental conditions, i.e. pristine ombrophilous dense forest, favour the existence of natural swamps and marshy aquatic ecosystems that could act as reservoirs for the microorganism. Regular agricultural activities, e.g., rice-field or banana production, in the vicinity of the forest remnants cause each year the transformation of the wet dense forest into an open-vegetation landscape, constituted by a mosaic of degraded forest patches, cultivated fields, fallows and degraded landscapes [40],[41]. Community encroachments and settlements in the vicinity of these patchy, altered environments may, thus, constitute foci for Buruli ulcer disease emergence and spread within human communities. On the contrary, in the South/South-East region of Côte d'Ivoire, where a vast coastal lagoon network, e.g. Ebrié, Aby, and two large dams, i.e. Ayamé 1 and 2, are associated with high human density settlements, Buruli ulcer disease is rare because coastal lagoons are brackish water ecosystems, thus potentially hostile to the development of *M. ulcerans* and/or its hosts/vectors, and man-made lakes are not associated with extensive agriculture on their shorelines. Finally, in the North of the country, small dams and rice fields are not associated to a high incidence of Buruli ulcer disease, although this area has known the development of the most important network of hydro-agricultural equipments in the last 20 years. The low rate of incidence recorded in the Northern area of Côte d'Ivoire can be best explained by the existence of a latitudinal limit possibly related to the bioclimatic North-South gradient. Indeed, contrary to the Southern region, due to the longer dry season period (more than 4 months) few dams and rivers are permanent throughout the year. This creates environmental conditions which are not favorable to the disease agent or its hosts/vectors persistence, thus, decreasing the contact opportunities for disease transmission to human populations. Indeed, the country-wide scale ecosystem signature of Buruli ulcer occurrence in Côte d'Ivoire, i.e. the highest incidence in the Centre of the country, where wet conditions are present all year around, in comparison to the North and the South, might be best explained by the existence of concomitant environmental factors, like the occurrence of specific biological species diversity that could be involved in the disease transmission.

The statistical regression analysis indicates that the most important risk factors for BU in human communities of Côte d'Ivoire are the irrigated rice crops (increase of risk by 77.5%). Other environmental factors, introduced into the analysis, are more marginal for the explanation of the disease risk: the interaction between dam surface and banana crops in the vicinity (increase of risk by 5.9%), and forest patch area and banana crops (increase of risk by 0.7%). We also showed that dam surfaces gave significantly lower odds for disease incidence whereas the increasing distance to dams was related to an increase in BU in Figure 5A. It seems that the distance to small dams is important for increased risk of BU infection, whereas the size of the dams is negatively related to the disease. This apparent discrepancy can be possibly explained by 2 main ways. First, this could be interpreted as a local-scale factor where small dams alter the environment in a way that is different from very large dams (similar to large lakes that usually do not have cases nearby). Small dammed areas can be much different in terms of the environment compared to large dams (lakes, reservoirs), and this is probably important to disease transmission and can imply different risk levels for BU. Here, we speculate that the risk of contamination is likely higher near small dams because of population behaviors. They used small dams for the irrigation of rice-field and banana crops. These small dams and irrigated crops are part of the everyday life of the neighbouring communities, and the dams constitute in most of cases, particularly in the Centre and North of Côte d'Ivoire, the only source of water supply for agro-pastoralism. Moreover, the potential interactions between primary forest patches and the development of agriculture may also constitute favorable ecotone zones that provide new environmental niches for the persistence and spread of *M. ulcerans*, and should be addressed.

To sum-up, it seems that the most important risk factor of BU regarding dams is not only its presence but mainly the contact between populations and these areas. To go further in this way and to be able to decide between (or define the proportion of) the two hypotheses discussed above (i.e. different environmental conditions and/or population behavior), field work is now required to determine the presence of the bacterium in environment and its potential different distribution in different areas (rice fields, small dams, large dams).

Thus, ecosystem dynamics and its evolution, e.g. land-use changes, biodiversity alteration, as well as socio-economic factors should be systematically taken into account in BUI research, putting this kind of study on a very promising way in order to better understand the transmission route of the bacterium from the environment to human populations, and then a better control of the disease. Further studies at different places and also at a multi-annual scale are now required to acquire a “bigger picture” of this disease.

All these findings are also consistent with two case-control studies in Benin and Ghana [19],[42] as well as a recent nation-wide study in Ghana [25], but they remain innovative since they define new risk factors (e.g. type of crops) for a new country, i.e. Côte d'Ivoire, and add socio-economic factors in the analyses. By extending the analysis of BU risk to a country-wide scale and to socio-economic factors, we highlight here, the importance of a multifaceted approach for disease surveillance. The ultimate goal of our research is to develop a quantitative, spatially realistic model for the BU system that will constitute the framework for the development of a sensible control plan. The present work demonstrates the importance of applying an environmental approach to the study of Buruli ulcer epidemiological problems and more generally highlights the strong necessity for an inter-disciplinary approach.

## Supporting Information

Alternative Language Abstract S1 Translation of the Abstract into French by Hélène Broutin
(0.03 MB DOC)

Alternative Language Abstract S2 Translation of the Abstract into Spanish by Hélène Broutin
(0.03 MB DOC)
