# Supplementary material for: Landscape Diversity Related to Buruli Ulcer Disease in Côte d'Ivoire
Source: PLoS Negl Trop Dis. 2008 Jul 30;2(7):e271. doi: 10.1371/journal.pntd.0000271 (PMC2474700; doi:10.1371/journal.pntd.0000271)
Supplement: Alternative Language Abstract S1 — Translation of the Abstract into French by Hélène Broutin (0.03 MB DOC) [file pntd.0000271.s001.doc]

L'Ulcère de Buruli (UB), dû à la bactérie *Mycobacterium Ulcerans*, est un important problème de santé publique émergent. Le mode de transmission à l'être humain de cette mycobactérie présente dans l'environnement n'est pas connu.

Méthodologie/résultats: nous avons étudié la relation entre les cas d'UB en Côte d'Ivoire, Afrique de l'Ouest, et un ensemble de variables environnementales, comme le type de végétation, la production agricole (riz et banane), les barrages et les lacs. Grâce à un système d'information géographique et à des analyses statistiques, nous mettons en évidence pour la première fois une relation à l'échelle nationale. Les rizières, et dans une moindre mesure les bananeraies, ainsi que la proximité des barrages d'irrigation, semblent augmenter le risque de contracter la maladie en Côte d'Ivoire. Cette association est particulièrement significative dans la région centrale du pays.

Conclusions: En accord avec diverses études cas-témoins antérieures, ce travail renforce l'identification de zones de risque élevé de contracter l'Ulcère de Buruli à l'échelle nationale. Cette première étude devrait être étendue à d'autres pays et à une échelle temporelle plus grande. Cela implique un effort considérable de collecte des données, pour obtenir une description globale des conditions environnementales qui conduisent à l'émergence et à la persistance de l'Ulcède de Buruli dans les populations humaines.

Mots-clés: Côte d'ivoire, Ulcère de Buruli, *mycobacterium Ulcerans*, environnement, émergence.

Translation by Hélène Broutin
