# Supplementary material for: Landscape Diversity Related to Buruli Ulcer Disease in Côte d'Ivoire
Source: PLoS Negl Trop Dis. 2008 Jul 30;2(7):e271. doi: 10.1371/journal.pntd.0000271 (PMC2474700; doi:10.1371/journal.pntd.0000271)
Supplement: Alternative Language Abstract S2 — Translation of the Abstract into Spanish by Hélène Broutin (0.03 MB DOC) [file pntd.0000271.s002.doc]

Contexto: La úlcera de Buruli (BUI), causada por la micobacteria *Mycobacterium ulcerans* representa un importante y emergente problema de Salud Pública, especialmente en varios países africanos. El modo de transmisión al ser humano de esta micobacteria que se encuentra en el medio ambiente falta ser elucidado.

Metodología/ Resultados Principales: En este estudio, hemos investigado la relación entre los casos de la úlcera de Buruli en la Costa de Marfil, África occidental, y un grupo de variables medioambientales tal como el tipo de vegetación, las cosechas (arroz y plátanos), las presas y los lagos. Utilizando un sistema de informaciones geográficas y análisis estadísticos hemos demostrado por primera vez una relación a la escala espacial nacional. Los arrozales, y en menor proporción los campos de plátanos y la proximidad de presas usadas para la irrigación y la acuacultura, parecen aumentar el riesgo de contraer la enfermedad en la Costa de Marfil. Este resultado es particularmente significativo en la región central del país.

Conclusiones/Importancias: Según lo sospechado ya por varios estudios caso-control anteriores ejecutados en varios países, nosotros consolidamos en este trabajo la identificación de áreas a riesgo elevado de contraer la úlcera de Buruli a la escala espacial nacional. Este primer estudio se debe ahora repetir en otros países y en una escala temporal más amplia. Eso implica un esfuerzo considerable en la colección y partición de datos para obtener una descripción global de las condiciones medioambientales que conducen a la aparición y la persistencia de la úlcera de Buruli en las poblaciones humanas.

**Key-words :** Costa de Marfil, úlcera de Buruli, *Mycobacterium ulcerans*, medio ambiente, emergencia.

Translation by Hélène Broutin
